# Genetic and phenotypic characterization of recently discovered enterovirus D type 111

**DOI:** 10.1371/journal.pntd.0007797

**Published:** 2019-10-17

**Authors:** Serge Alain Sadeuh-Mba, Marie-Line Joffret, Arthur Mazitchi, Marie-Claire Endegue-Zanga, Richard Njouom, Francis Delpeyroux, Ionela Gouandjika-Vasilache, Maël Bessaud

**Affiliations:** 1 Virology Service—Centre Pasteur of Cameroon–Yaounde, Cameroon; 2 Institut Pasteur—Unité de biologie des virus entériques—Paris, France; 3 WHO Collaborating Centre for Enteroviruses and Viral Vaccines—Paris, France; 4 Enteric Viruses and Measles Laboratory—Institut Pasteur de Bangui—Bangui, Central African Republic; Faculty of Science, Ain Shams University (ASU), EGYPT

## Abstract

Members of the species *Enterovirus D* (EV-D) remain poorly studied. The two first EV-D types (EV-D68 and EV-D70) have regularly caused outbreaks in humans since their discovery five decades ago but have been neglected until the recent occurrence of severe respiratory diseases due to EV-D68. The three other known EV-D types (EV-D94, EV-D111 and EV-D120) were discovered in the 2000s-2010s in Africa and have never been observed elsewhere. One strain of EV-D111 and all known EV-D120s were detected in stool samples of wild non-human primates, suggesting that these viruses could be zoonotic viruses. To date, EV-D111s are only known through partial genetic sequences of the few strains that have been identified so far. In an attempt to bring new pieces to the puzzle, we genetically characterized four EV-D111 strains (among the seven that have been reported until now). We observed that the EV-D111 strains from human samples and the unique simian EV-D111 strain were not phylogenetically distinct, thus suggesting a recent zoonotic transmission. We also discovered evidences of probable intertypic genetic recombination events between EV-D111s and EV-D94s. As recombination can only happen in co-infected cells, this suggests that EV-D94s and EV-D111s share common replication sites in the infected hosts. These sites could be located in the gut since the phenotypic analysis we performed showed that, contrary to EV-D68s and like EV-D94s, EV-D111s are resistant to acid pHs. We also found that EV-D111s induce strong cytopathic effects on L20B cells, a cell line routinely used to specifically detect polioviruses. An active circulation of EV-D111s among humans could then induce a high number of false-positive detection of polioviruses, which could be particularly problematic in Central Africa, where EV-D111 circulates and which is a key region for poliovirus eradication.

## Author summary

Many examples of emergence of viruses that trigger severe diseases in humans are known. Emergence can be due to the sudden increase of the pathogenic power of a virus that had silently circulated into human populations for a long period; it can also be due to the cross-species transmission of a virus from its animal host to humans. The recent outbreaks of severe respiratory diseases due to enteroviruses D68 (EV-D68) brought to the light to potency of members of the species *Enterovirus D* (EV-D) to emerge as severe human pathogens.

By many aspects, EV-Ds are still mysterious: their natural history and epidemiology are poorly studied and even their main hosts remain unknown. For decades, EV-Ds were believed to infect mainly humans but recent data about EV-Ds identified in sub-Saharan Africa support their zoonotic origin. In an attempt to increase our knowledge about EV-Ds, we undertook genetic and phenotypic characterization of four EV-D111 isolates, a virus type that was recently uncovered in humans and in non-human primates in Central Africa. Our results show that EV-D111s are probably enteric viruses and evolve by exchanging genetic sequences with EV-D94.

## Introduction

Enteroviruses (EVs) are small naked viruses belonging to the family *Picornaviridae* [1]. Their genome consists of a unique molecule of single-stranded RNA of about 7,500 nucleotides (nt) in length. This genome is positively stranded, *i*.*e*. it is directly used as a messenger RNA by the cellular machinery. It contains a large open reading frame (ORF) that is translated into a unique polyprotein, which is subsequently cleaved to give rise to the functional proteins. The 5’ part of the ORF encodes the four proteins that form the capsid (VP1 to VP4); downstream, the genome encodes different proteins that are involved in the virus cycle.

Since the particles are naked, the capsid determines important features of the circulating EVs, such as their tropism or their immunogenicity. Consequently, the classification of EVs mainly relies on the capsid-encoding genomic sequences. To date, the International Committee for Virus Taxonomy has defined 15 species [2, 3]. Among these species, the species *Enterovirus A* to *C* and *Rhinovirus A* to *C* are the best known because their members mainly infect humans. Each of these species contains tens of types.

By contrast, only five types have been described within the species *Enterovirus D* (EV-D). Two types, EV-D68 and EV-D70 were identified decades ago and have been regularly observed worldwide. Discovered in 1962 in California, EV-D68 shares properties with respiratory viruses and was responsible for large outbreaks of severe respiratory illnesses in the 2010s [4]. Identified at the beginning of the 1970s, EV-D70 has an ocular tropism and is one of the main etiologic agents of haemorrhagic conjunctivitis [5]. The three other known EV-D types (EV-D94, -111 and -120) were identified more recently and were observed exclusively in Africa. The first EV-D94 was isolated from stool samples collected in Democratic Republic of the Congo and from sewage specimens collected in Egypt [6, 7]; EV-D120s were detected in few faecal samples of non-human primates (NHPs) living in Cameroon [8] but none EV-D120 strain has ever been isolated in cell culture.

As for EV-D111, the first isolate was recovered from human stool samples collected in Democratic Republic of the Congo but it was misidentified as EV-D70 [6]. Another member of this type was subsequently detected by molecular methods in a stool sample of a wild chimpanzee living in the Cameroonian forest [9]. Later, other EV-D111s were isolated from human stool samples collected in Central African Republic and in Cameroon [10, 11]. To date, this type is only known by partial genomic sequences. In an attempt to increase our knowledge about members of the species *Enterovirus D*, we undertook the sequencing of the whole genomes of four EV-D111 isolates belonging to our collection and conducted further biological characterization. Our results show that, contrary to EV-D68s and like EV-D94s, EV-D111s are acid-resistant, which make them able to replicate in the gut. Our results also suggest that EV-D94s and EV-D111s probably co-evolve through intertypic genetic recombination.

## Methods

### Viruses and cells

The four EV-D111s had been isolated in cell cultures from stool samples of children with acute flaccid paralysis in the framework of poliovirus surveillance [10, 11]. The strains Fermon, J670/71 and Nancy were isolated decades ago and are the prototype strains of the types EV-D68, EV-D70 and coxsackievirus B3 (CV-B3), respectively; they are available through the European Virus Archive [12]. The poliovirus type 1 (PV-1) and type 3 (PV-3) vaccine strains Sabin belong to the collection of our laboratory. All works with infectious materials were done in a BSL-2 facility.

HEp-2c and RD cells were provided by the US CDC (Atlanta, USA) and L20B cells were provided by the National Institute for Biological Standards and Control (NIBSC, Potters Bars, UK). MA104, LLC-MK2 and C6/36 cells were from the ATCC. Murine L cells are from the Institut Pasteur’s collection. Cell culture media are described in details in S1 Table.

### Sequencing and sequence analysis

Viral RNAs were extracted from cell culture supernatants with the High Pure Viral RNA kit (Roche Diagnostics). Virus genomes were amplified by RT-PCR with generic primers as previously described [13]: briefly, for each virus, this amplification step leads to the generation of two large DNA products that together span the whole genome; these two products were mixed together and sequenced on an Illumina platform as already described [13]. The reads were trimmed and used for *de novo* assembly of the genomes using CLC Genomics Workbench 9.0 with parameters already optimized [14].

Multiple sequence alignments were performed with CLC Main Workbench 8 software (CLC bio) and phylograms were constructed with the MEGA 5 program [15]. Similarity plots were drawn with the SimPlot software [16] using the Hamming distance method (200 nt-wide sliding window, step size of 20 nt).

### Acid sensitivity assay

The acid sensitivity assay was performed on EV-D111 OUP-05-059, using EV-D68 Fermon and EV-D70 J670/71 (known to be acid sensitive and acid resistant, respectively [17]) as controls. Three hundred microlitres of viral stocks were mixed with 300 μL of 0.5 M citrate buffer pH 3.0 or 0.1 M phosphate buffer pH 7.2. The mixtures were incubated 1 h at 37°C and then neutralized by adding 600 μL of 0.1 M phosphate buffer pH 7.2 and 1.8 mL of Minimum Essential Medium medium. The neutralized solutions were titrated on RD cells according to the WHO standard protocol and using the Spearman-Kärber method [18].

### Infection assay

The ability of EV-D111 OUP-05-059 to produce cytopathic effect in different cell lines was assessed in 96-well plates. The cells were trypsinized and resuspended in fresh culure medium at a concentration of 1.2x10^5^ cells per mL; the suspensions were used to seed 96-well plates (150 μL per well). Ten-fold dilutions (from 10^0^ through 10^−7^) of a viral stock were added into the wells (50 μL per well, 10 wells per dilution). The same virus solutions were used to inoculate all cell lines; therefore, for a given dilution, all cell lines were inoculated with the same number of infectious particles. The plates were incubated 6 days at 37°C under 5% CO_2_ (except for C6/36 cells: 28°C without CO_2_) and were microscopically examined to detect cytopathic effects.

To determine whether EV-D111 grows in L cells, L cells were cultivated in 24-well plates until confluence. One hundred microlitres of virus suspensions were added to each well and plates were incubated at 37°C for 1 hour. After incubation, the inoculum was removed and 1 mL of fresh medium was then added into each well. One plate was immediately frozen at -20°C and two others were incubated at 37°C for 2 days or for 5 days before freezing. The three plates were frozen and thaw three times and well contents were collected and clarified by centrifugation. The solutions thus obtained were titrated on RD cells according to the WHO standard protocol and using the Spearman-Kärber method [18].

### Poliovirus molecular screening

In order to detect a putative contamination of the EV-D111 OUP-05-059 stock solution by a poliovirus, the supernatant of a positive L20B well was screened with the WHO Poliovirus rRT-PCR ITD 5.0 kit [19] using the Quanta ToughMix kit.

### Production of anti-CD155 monoclonal antibody

Hybridoma D171 was kindly provided by Günter Bernhard (Institute of Immunology, Hannover Medical School, Hannover, Germany). This hybridoma was grown in RPMI 1640 supplemented with 2 mM glutamine and 10% foetal calf serum (S1 Table). For antibody production, the cells were harvested by centrifugation and resuspended in RPMI 1640 supplemented with 2 mM glutamine and 2% foetal calf serum and incubated for one week at 37°C under 5% CO_2_. The cell culture supernatant was then clarified by centrifugation and used for immunofluorescence and infectivity inhibition assays.

### Indirect immunofluorescence assay

The ability of antibody D171 to bind cells was checked through an indirect immunofluorescence assay with no fixation step [20, 21]. Briefly, L20B, RD or L cells were grown in 24-well plates. Cell culture supernatant was aspirated and cells were incubated for 1 h with 500 μL of phosphate buffer saline (PBS) supplemented with 10 μg.mL^-1^ bovine serum albumin (BSA) to block unspecific binding of the antibodies. The blocking solution was removed and cells were incubated for 1 h with 500 μL of undiluted supernatant of D171 hybridoma culture. After two consecutive washes with 1.5 mL PBS, cells were incubated 1 h with the secondary antibody (Invitrogen Goat anti-Mouse IgG (H+L) Cross-Adsorbed Secondary Antibody, Alexa Fluor 488, reference A-11001) diluted in PBS supplemented with 10 μg.mL^-1^ BSA. Cells were observed on an inverted fluorescence microscope.

### Infectivity inhibition assay with anti-CD155 monoclonal antibody

The protecting activity of antibody D171 was assessed following a technique already reported [22]. Briefly, RD or L20B cells grown in flasks were trypsinized and used to seed 96-well plates (100 μL per well) at a concentration of 1.8x10^5^ cells per mL in Minimum Essential Medium Eagle supplemented with 4 mM glutamine and 4% foetal calf serum. Each well was supplemented with 50 μL of a solution containing 20 μL of D171 hybridoma supernatant and 30 μL of Minimum Essential Medium Eagle; in control plates, wells were supplemented with 50 μL of Minimum Essential Medium Eagle (no antibody). The plates were incubated 90 min at 37°C under 5% CO_2_.

Ten-fold serial dilutions of virus in Minimum Essential Medium Eagle were then added (50 μL per well, 10 wells per dilution) and the plates were incubated 5 days before reading of the virus titre.

## Results

### De novo assembly of EV-D111 full-length genomes

Three isolates sequenced during this work (OUP-05-059, NGB-06-107 and BAN-06-150) were by-products of the poliovirus surveillance and were isolated from human stool samples collected in Central African Republic in 2005 and 2006 [10]; the fourth isolate (TOK-230) came from a stool sample of a healthy child collected in Cameroon in 2008 within the framework of an epidemiological study [11]. All were previously identified as EV-D111s by a typing method based on the VP1-encoding sequence [23, 24] but no other genetic data were available for these isolates. In this work, the whole genome of the four isolates was amplified by using EV generic primers that generated two overlapping amplicons that were subsequently sequenced on an Illumina platform. For all four samples, *de novo* assembly gave rise to a contig spanning the complete genome of the studied EV-D111, except few 5’ and 3’ terminal nt that correspond to the RT-PCR primer binding sites. In the VP1-encoding region, the contigs generated during this study did not differ by more than 3 nt from the sequence previously obtained from the same sample through the Sanger sequencing technique [10, 11].

Two supernatants were found to contain a mixture of viruses: besides the expected EV-D111, BAN-06-150 contained an echovirus 19 (E-19, Genbank accession number MK032893) and TOK-230 contained an E-19 (Genbank accession number MK032892) and a coxsackievirus B1 (CV-B1, Genbank accession number MK032894). These viruses belong to the species *Enterovirus B* and had not peculiar genetic relationships with EV-Ds in any parts of their genomes.

**Fig 1:**
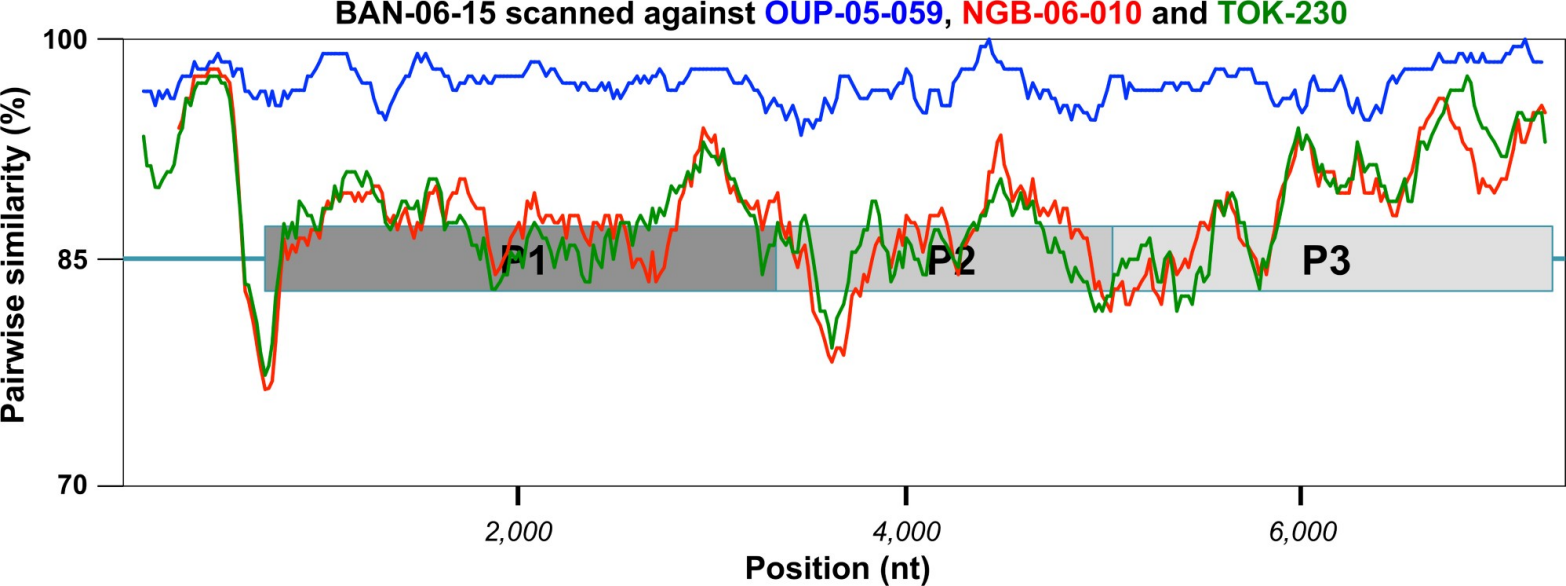
Comparative analysis of the four EV-D111 full-length genomes. A similarity plot was drawn by comparing the genomic sequence of the strain BAN-06-150 with those of the four other EV-D111 strains sequences in this study.

The four EV-D111 genomes had the canonical organization of the EV genomes. They had a 6,582 nt ORF flanked by two untranslated sequences. The 5’UTR and 3’UTR length ranged from 724 to 728 nt and from 51 to 54 nt, respectively (without the nt targeted by the RT-PCR primers). Altogether, the four viruses displayed a similarity ≥ 88.2% at nt level (Table 1). The two isolates OUP-05-059 and BAN-06-150 displayed the closest sequence match along the genome (Fig 1) and featured a global nt similarity of 97.0% between each other. The polyprotein sequence was highly conserved among the four EV-D111s, with a peptidic divergence < 3% (*i*.*e*. less than 60 amino-acid (aa) differences over the 2,194 aa-long polyprotein).

**Table 1:** Pairwise identity between the genomes of the studied EV-D111 isolates (lower left) and between the deduced polyprotein (upper right).

	NGB-06-107	TOK-230	BAN-06-150	OUP-05-059
**NGB-06-107**		98.5%	97.3%	97.5%
**TOK-230**	94.5%		97.3%	97.5%
**BAN-06-150**	88.2%	88.2%		99.0%
**OUP-05-059**	88.6%	88.7%	97.0%	

**Fig 2:**
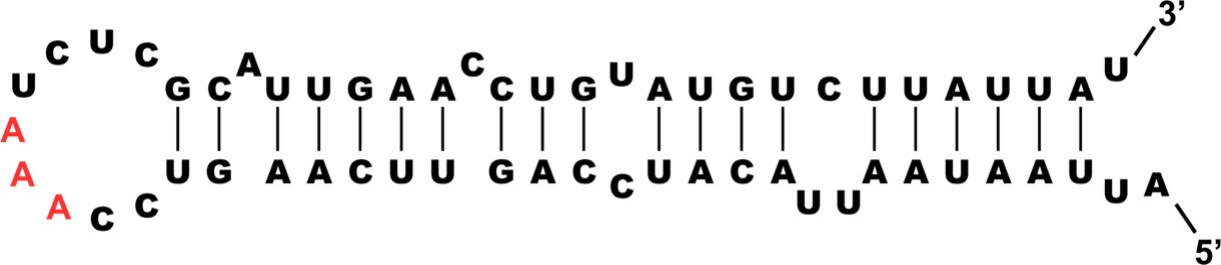
Predicted secondary structure of the cis-acting replicating element of EV-D111. The triplet known to be directly involved in the uridylation of the protein 3B is in red.

The putative polyprotein cleavage sites were identified by comparison with those of other EVs. They were conserved among the four viruses and most cleavage sites have a glutamine residue at position P1 (according to the nomenclature of protease substrate residues [25]) (S2 Table). The 2C-encoding region contained a 58 nt-long stretch perfectly conserved among the four EV-D111 sequences. This stretch contained the GNNNAAANNNNNNA motif known to be a cis-replicating acting element, which is indispensable for EV replication [26]. Different models predicted that this stretch forms a hairpin structure with a loop containing the AAA triplet known to be directly involved in the uridylation of the protein 3B [27] (Fig 2).

**Fig 3:**
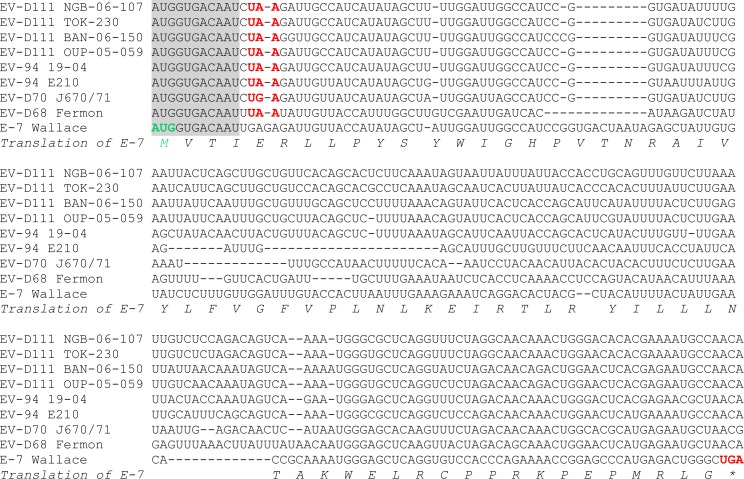
Multiple alignment of genomes of EV-Ds and echovirus 7 in the region of the recently discoverd short open reading frame. The alignment starts at nucleotide position 591 of the E-7 strain Wallace genome. The uORF start codon is highlighted in green; the in-frame stop codons are highlighted in red. The 11 nt-long sequence perfectly conserved at the beginning of the uORF is shaded in grey.

Recently, a short additional ORF (so-called uORF) has been described within the genome of EV-As, Bs and Cs [28]: the uORF starts inside the domain VI of the internal ribosome entry site and ends in the VP4 encoding-sequence; thus, the uORf and the large ORF overlap each other but use different reading frames. In order to check whether EV-D111 genomes harboured an ORF similar to the short uORF that was confirmed in the genome of the E-7 prototype strain, a multiple alignment was constructed (Fig 3). Interestingly, the very beginning of the E-7 uORF, including the start codon, is perfectly conserved in EV-D genomes. Nonetheless, while the E-7 uORF extends on 201 nt, all EV-D genomes featured an in-frame stop codon located few nt downstream the conserved ATG. Thus, the EV-D uORF can only encode four amino-acids and is probably not functional.

### Phylogenetic relationships with other EVs

**Fig 4:**
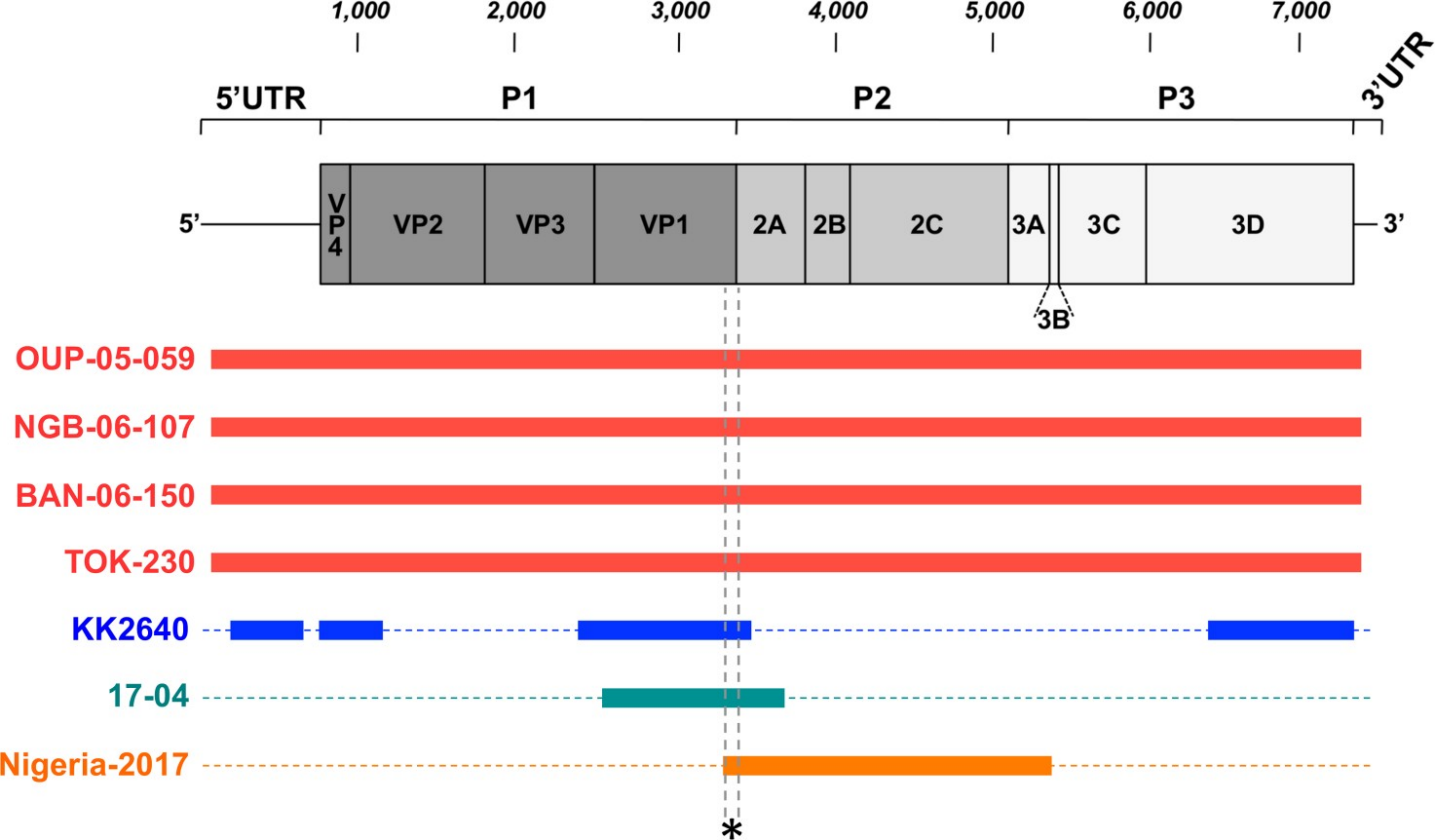
Schematic representation of the genetic data available for the known EV-D111 strains. Solid bars cover the genomic regions whose sequences are available. The asterisk indicates the short region used to draw the phylogram featured as S1 Fig.

The genetic sequences of the four isolates were compared to those of other EV-D111s available in public database. Actually, very few EV-D111s have been detected or isolated and only partial sequences are available (Fig 4). KK264 genome was sequenced in the 5’UTR and the VP4-VP2, the VP1 and the 3D regions whereas only the VP1-2A region of 17–04 genome has been published. More recently, the partial sequence of an EV-D111 isolated in Nigeria was released in GenBank: this sequence covers the genome from the very end of VP1 to the beginning of the 3C.

**Fig 5:**
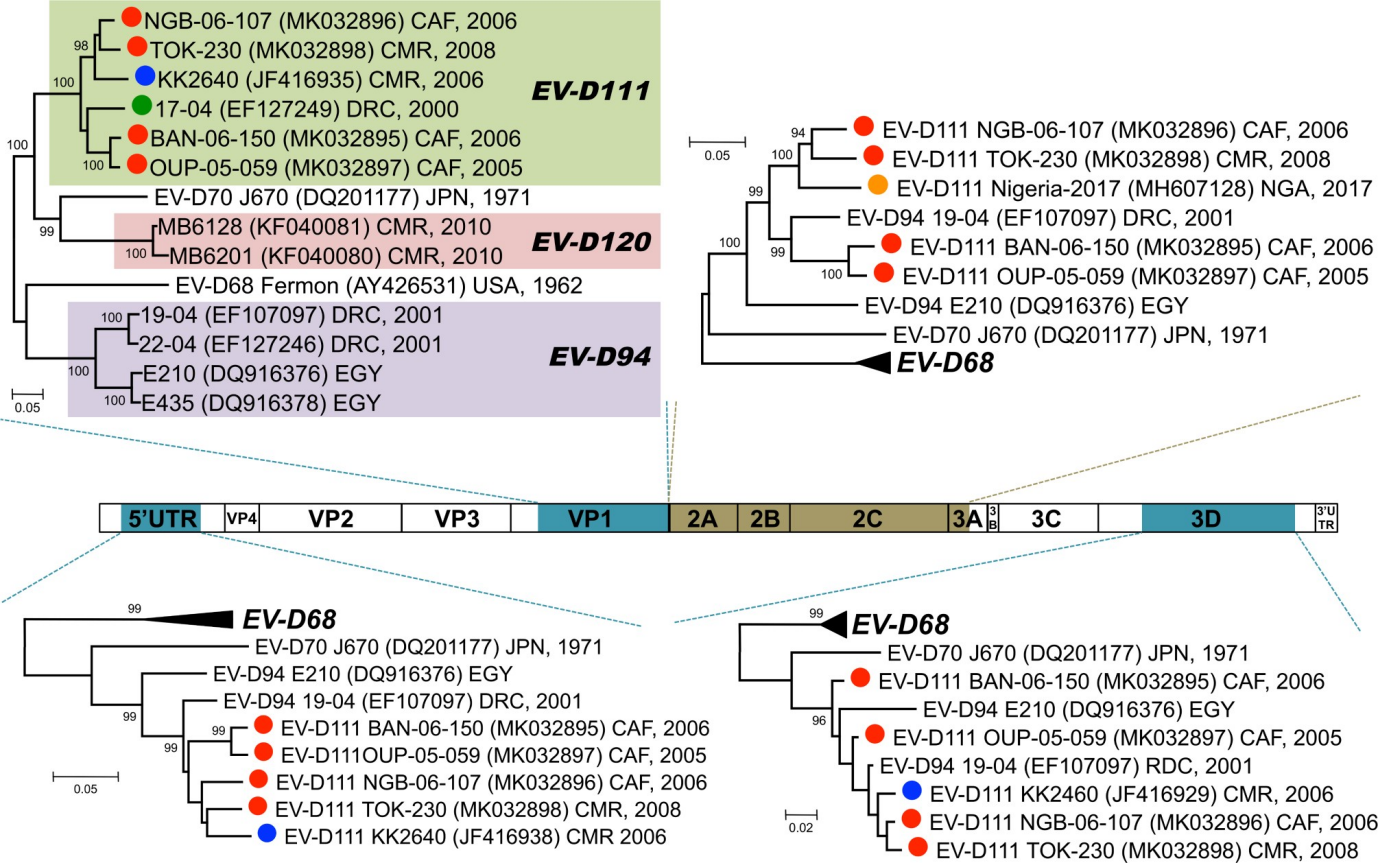
Phylogenetic trees based on different genomic regions. The organization of the enterovirus genome is schematized in the middle of the picture; the genomic regions used to draw the phylogenetic trees are shaded. In the trees, the EV-D111 labels are colour-coded according the colours used in Fig 4.

In the VP1 region, the EV sequences cluster according to their type. In this region, the 6 EV-D111 strains for which a substantial part of the VP1 sequence is available (*i*.*e*. all except Nigeria-2017) formed together a group supported by a 100% bootstrap value (Fig 5). Inside this group, the clustering was similar to that previously observed based on partial VP1 sequences [11]. It is of note that the EV-D111 strain originating from a simian sample (KK2640) in the one hand and the EV-D111 strains from human samples in the other hand did not form two separate phylogenetic lineages: NGB-06-107, TOK-230 and KK2640 [9] formed together a branch supported by a high bootstrap value (98%) whereas 17–04 occupied an intermediate position between this group and the one defined by isolates BAN-06-150 and OUP-05-059. We included the Nigeria-2017 partial VP1 sequence in our alignment and drew the corresponding phylogram. Although this phylogram gathered all the known EV-D111 strains, it was poorly informative since the bootstrap values were very low, even for the EV-D111 group (S1 Fig); these low values are due to the shortness of the region where KK2640 and Nigeria-2017 sequences overlap together (167 nt in length, Fig 4).

Interestingly, EV-D111 and EV-D120 sequences do not cluster with those of EV-D94s in the VP1 region: in this region, EV-D70, EVD111 and EV-D120 sequences formed together a group supported by a 100% bootstrap value (Fig 5).

**Fig 6:**
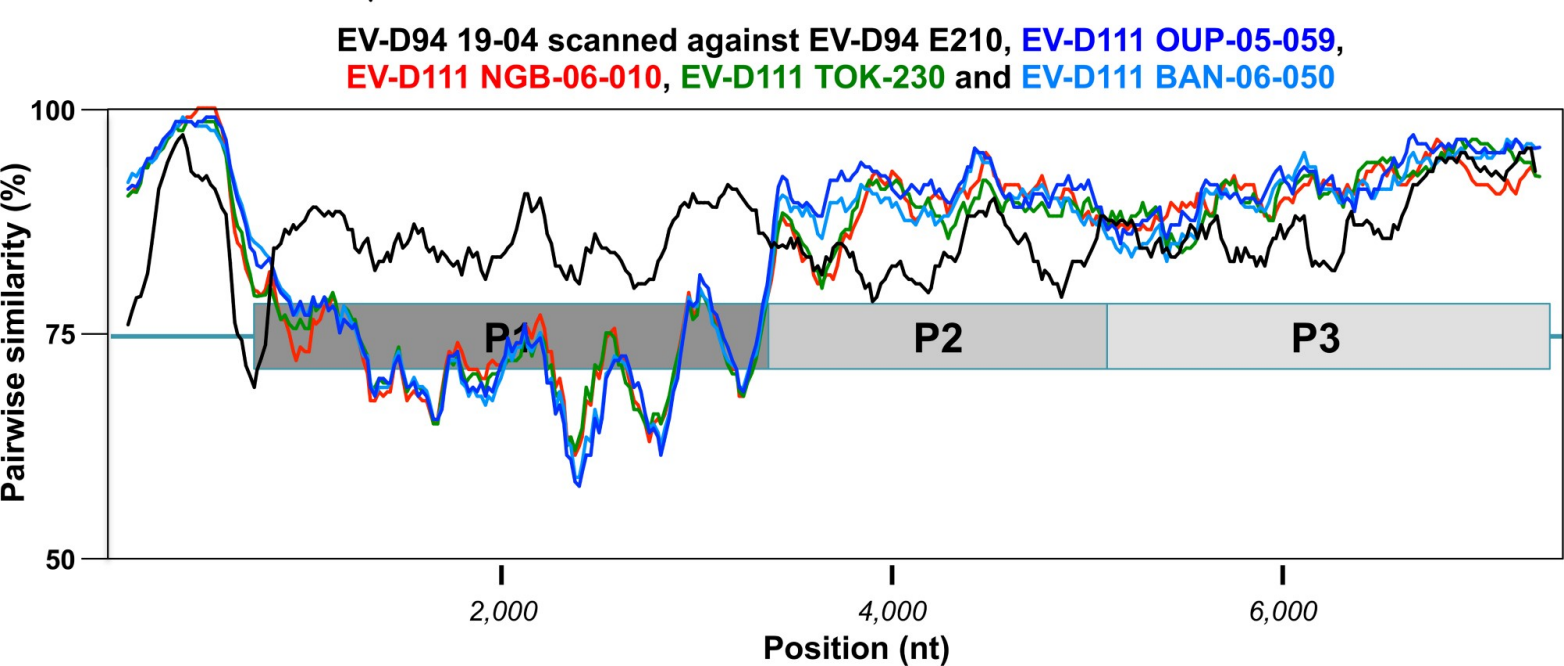
Comparative analysis of EV-D94 and EV-D111 full-length genomes. A similarity plot was drawn by comparing the genomic sequence of the strain EV-D94 19–04 with those of other EV-Ds.

We also investigated the relationships of EV-D111 strains together and with other EV in other genomic regions. The nt sequences corresponding to the regions 5′UTR, 2A, 2B, 2C, 3A, 3B, 3C, and 3D of the genome were used individually in separate BLAST analyses to identify, for each region, the closest sequences available in the GenBank database. In all the genomic regions, the closest sequences were from viruses belonging to the other EV-D types, particularly EV-D94. In the 5’UTR, the P2 and the 3D regions, the few known EV-D94 and EV-D111 sequences clustered together (bootstrap values ≥96%) and no EV-D68 or EV-D70 sequences were found particularly related to the EV-D94/EV-D111 cluster (Fig 5). In these three regions, EV-D94 sequences and EV-D111 sequences did not form two separate clusters. A similarity plot analyses conducted along the complete genome showed that, except in the capsid-encoding region, EV-D94 strain 19–04 was even closer to EV-D111s than to the homotypic strain E210 (Fig 6).

### Phenotypic properties

As explained above, the viral stocks of strains TOK-230 and NGB-06-107 contained other EVs. Besides, the viral stock of BAN-06-150 had lost its infectivity, probably because of previous storage under inappropriate conditions. Therefore, the phenotypic characterization was conducted using the strain OUP-05-059.

**Fig 7:**
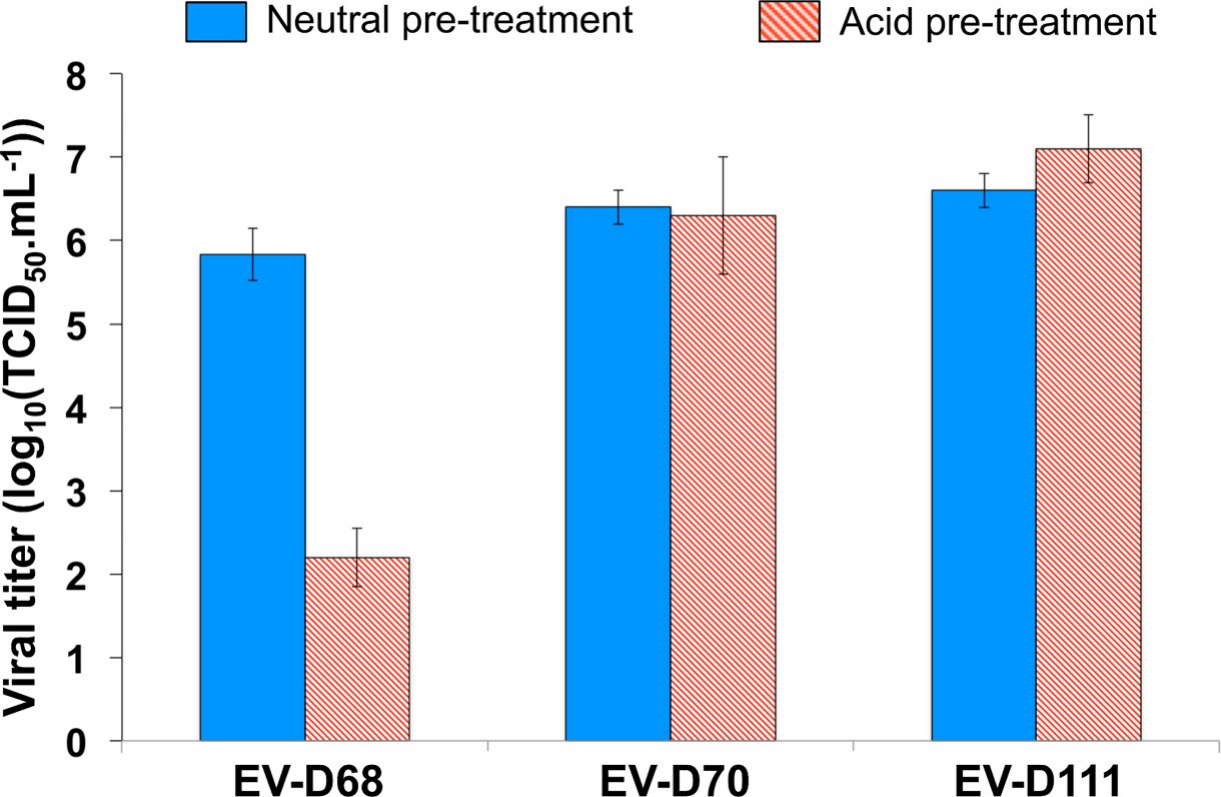
Acid-sensitivity assay. EV-D68 strain Fermon, EV-D70 strain J670/71 and EV-D111 strain OUP-05-059 suspensions were incubated at neutral or acid pH before being titrated on RD cells. The error bars indicate the 95% confidence intervals calculated on triplicates.

EV-D68 is acid-sensitive while EV-D94 is not. In order to determine whether the acid-sensitive phenotype of EV-D68 is the exception rather than the rule among EV-Ds, we determined whether the infectivity of EV-D111 is altered by low pHs. For this purpose, an infectivity assay was conducted on RD cells with or without acid pre-treatment of the viruses. While acid pre-treatment led to a decrease of more than 3 log_10_ of the EV-D68 infectivity, no change was observed for EV-D70 and EV-D111 (Fig 7).

**Fig 8:**
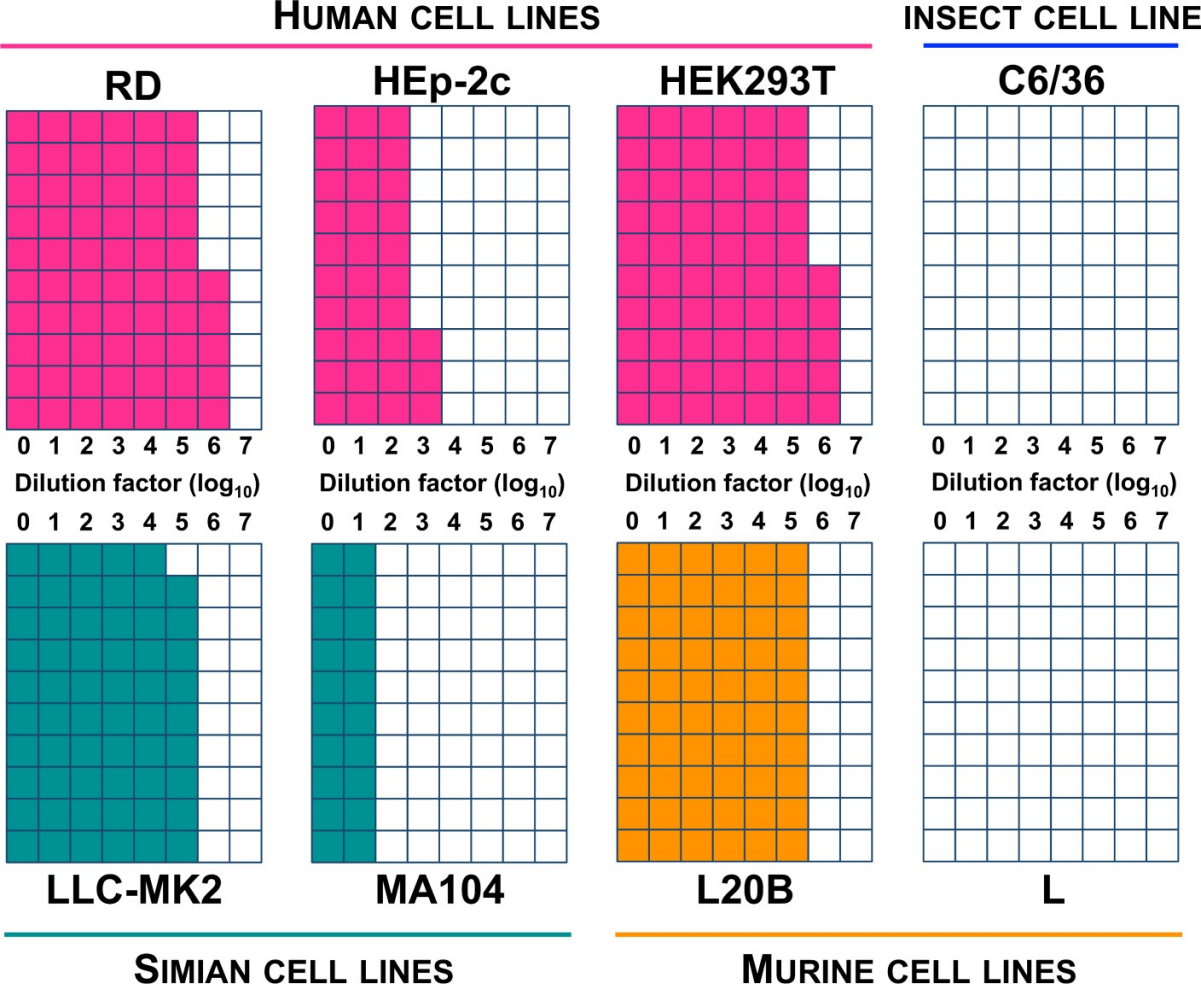
Susceptibility of different cell lines to EV-D111 strain OUP-05-059. Serial 10-fold dilutions of a virus suspension were used to infect cells in 96-well plates (10 wells per dilution for each cell line). Cell monolayers were checked microscopically 6 days post-infection to detect cytopathic effects. For each dilution, the coloured squares indicate the number of wells in which cytopathic effects were detected.

The ability of EV-D111 to induce cytopathic effects was assessed on different common cell lines. Cytopathic effects were observed on RD and HEp-2c, two human cell lines commonly used to isolate human enteroviruses. However, HEp-2c cells were found substantially less sensitive compared to RD cells (Fig 8). As the EV-D111 isolate used in this experiment had been passaged several times on RD cells, we assessed whether the relatively high sensitivity of RD could be due to an adaptation of the virus to this particular cell line. For this purpose, we evaluated the sensitivity of another human cell line, HEK293T; we found that its sensitivity was similar to that of the RD cells.

As an EV-D111 was detected in a chimpanzee stool sample [9], we also evaluated the sensitivity of two simian kidney cell lines, LLC-MK2 and MA104, that were previously used to grow different types of simian enteroviruses [29, 30]. Cytopathic effects were observed on both cell lines but MA104 were poorly sensitive compared to LLC-MK2 (Fig 8).

EV-D111 also induced cytopathic effects on L20B, a cell line commonly used by the Global Polio Laboratory Network to isolate poliovirus from stool and environmental samples. The kind of cytopathic effects observed in L20B cells infected by EV-D111 was similar to that induced by polioviruses, with the total destruction of the monolayer after a few days. The L20B cell line derives from L cells that were transfected with the gene of the human poliovirus receptor, CD155 [31]. We found that EV-D111 does not induce cytopathic effects on L cells, even when these cells are inoculated with undiluted EV-D111 cell culture supernatant (Fig 8). In order to determine whether the cytopathic effects observed on L20B could be due to a contaminating poliovirus, a poliovirus-targeting molecular assay was performed on the supernatant of a positive L20B well. This assay gave a negative result. An infectivity inhibition assay was also conducted by using the D171 monoclonal antibody. This antibody targets CD155 and was selected for its protection of HeLa cells against the cytopathic effects of PV-1 [22]. The ability of our antibody solution to bind CD155 was assessed by immunofluorescence. As expected, fluorescent staining was observed on L20B and RD monolayers while no staining was observed on L cells (S2 Fig).

**Fig 9:**
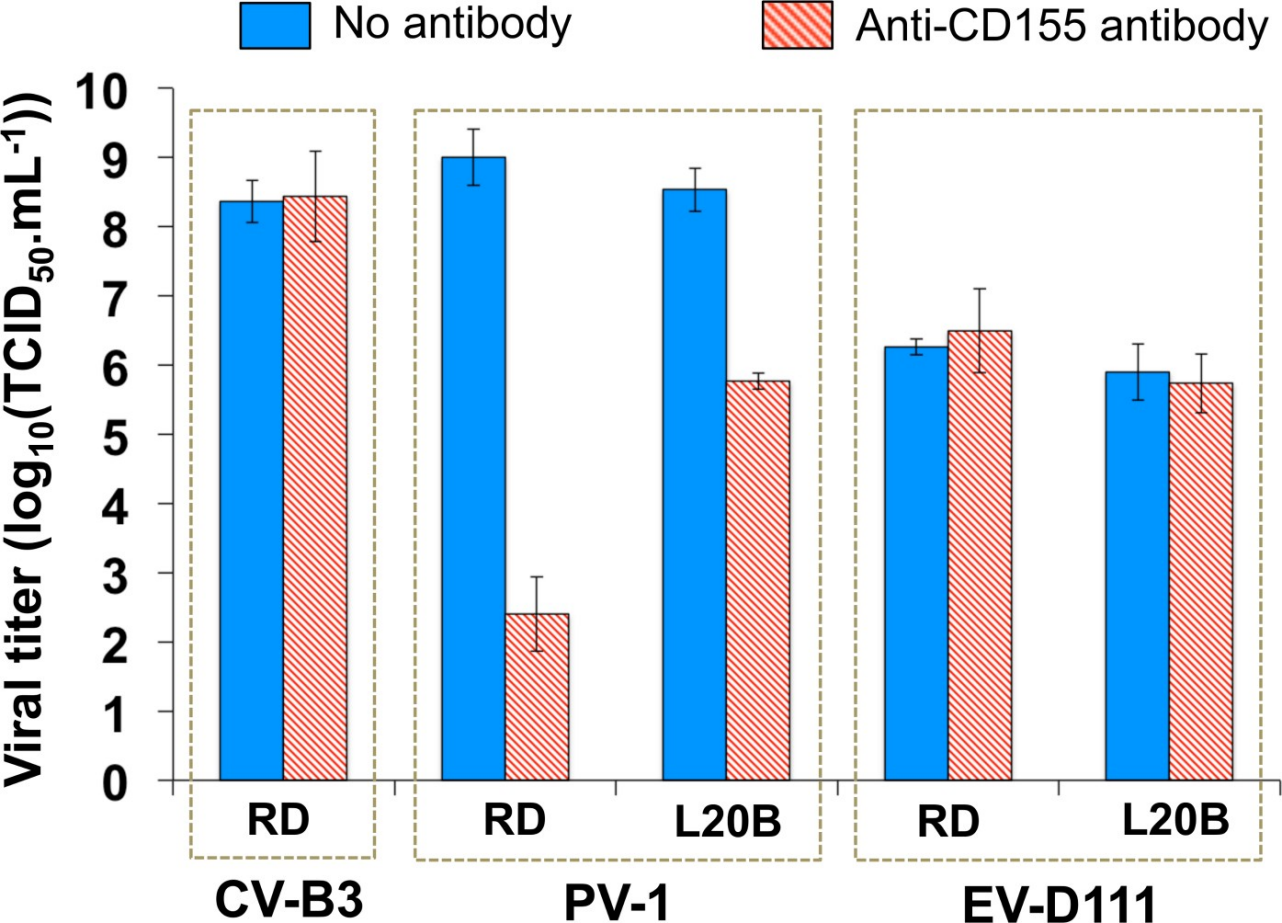
Protection of cells by antibody D171 against virus-induced cytopathic effect. Cells seeded in 96-well plates were pre-incubated with or without antibody D171. Plates were than used to titrate CV-B3 strain Nancy, PV-1 strain Sabin and EV-D111 strain OUP-05-059 suspensions. The error bars indicate the 95% confidence intervals calculated on triplicates.

D171 reduced substantially the infectivity of a PV-1 cell culture supernatant: the virus titre decreased about 3 log_10_ on L20B and about 6 log_10_ on RD (Fig 9). No decrease was observed in the case of CV-B3, a virus that does not use CD155 as receptor; this result confirmed the specificity of the inhibition due to D171. No inhibition was observed for the EV-D111 supernatant. This result and the negative result obtained through the poliovirus-targeting molecular assay were consistent together and led us to the conclusion that the cytopathic effect detected on L20B cells was not due to the presence of a contaminating poliovirus.

**Fig 10:**
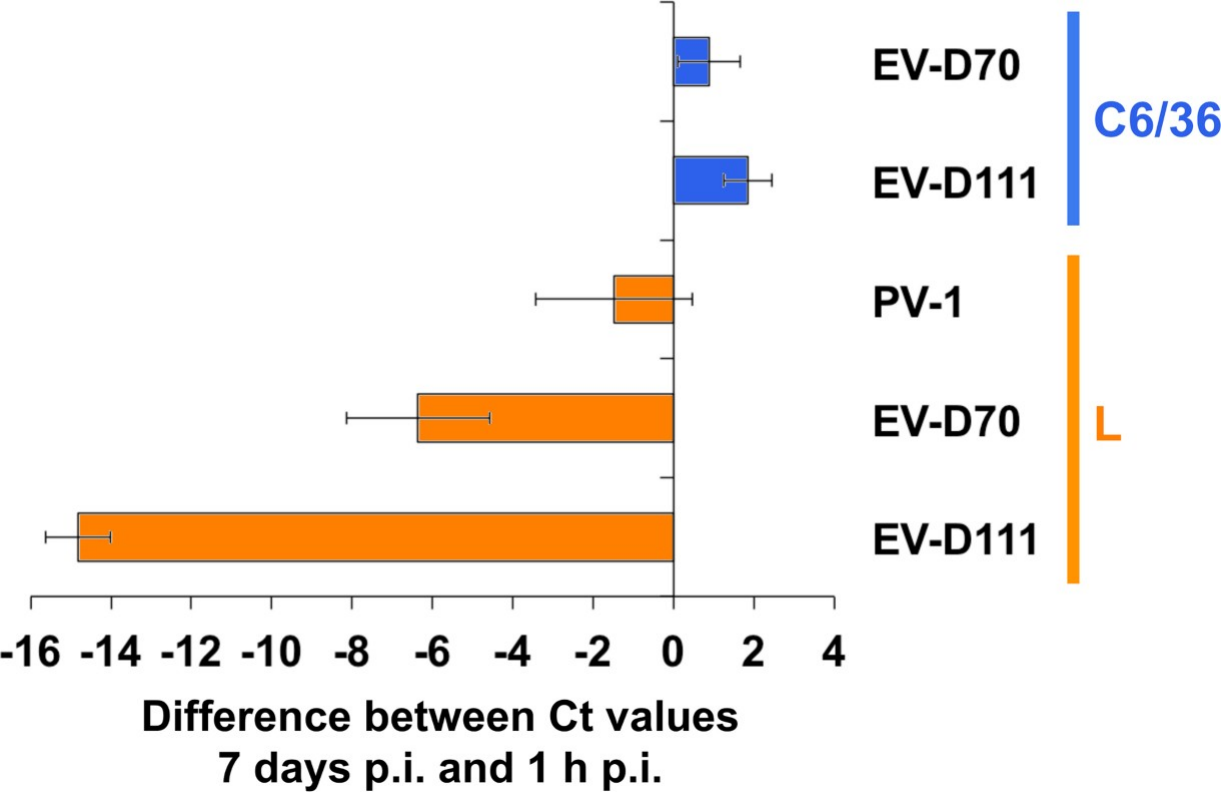
Detection of virus replication in L and C6/C6 cells. PV-1 strain Sabin, EV-D70 strain J670/71 and EV-D111 strain OUP-05-059 suspensions were used to inoculate L cell monolayers; C6/36 monolayers were inoculated with EV-D70 and EV-D111 only. RNA was extracted from clarified supernatant 1 hour post-inoculation and 7 days post-inoculation and EV RNA was detected through real-time RT-PCR. Virus replication leads to an increase of the viral RNA concentration during the course of time and to a drop of cycle threshold (Ct) values. Therefore, negative values on x-axis reveal virus replication.

Previously, EV-D70 was found able to grow in L cells without inducing any visible cytopathic effect [32]. In order to check whether, similarly, EV-D111 was able to replicate into these cells, L cell monolayers were incubated with EV-D111, EV-D70 and PV-1 suspensions. Viral RNA was detected by real-time RT-PCR 1 hour and 7 days after inoculation. As expected, no significant shift in Ct values was observed for PV-1 between the two incubation times (Fig 10), in accordance with the fact that this virus is not able to infect L cells. By contrast, clear shifts were observed for both EV-D70 and EV-D111, indicating that these two viruses are able to infect this cell line and replicate their genomes.

**Fig 11:**
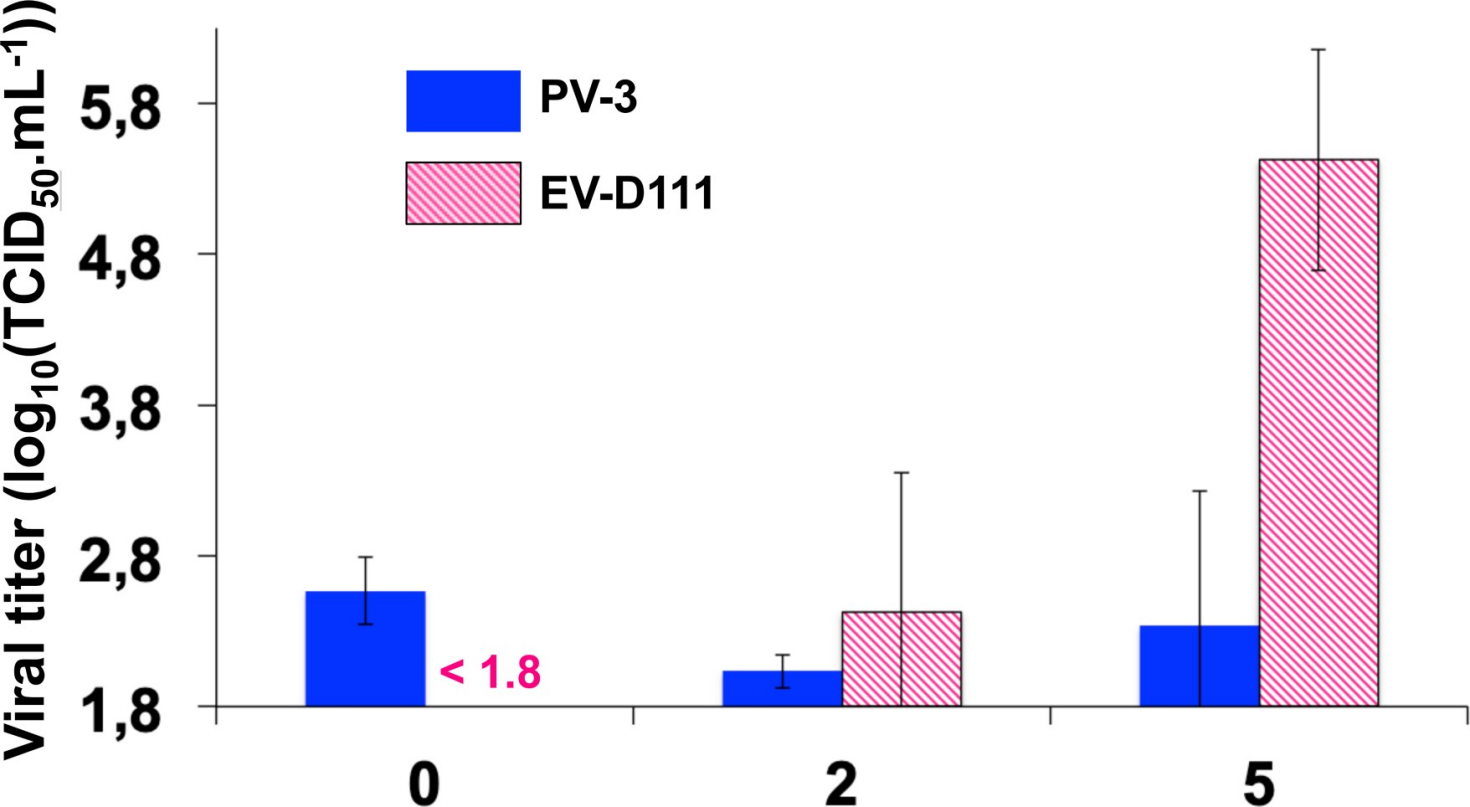
Replication kinetics assay in L cells. PV-3 strain Sabin and EV-D111 strain OUP-05-059 suspensions were used to inoculate L cell monolayers (1,000 TCID_50_ and 100 TCID_50_ per well, respectively). After three freeze-thaw cycles, well contents were harvested and clarified, and viruses were titrated on RD cells. Errors bars indicate the 95% confidence interval calculated on 4 wells. Titres lower than 101.8 TCID_50_.mL^-1^ cannot be measured.

To determine whether EV-D111 infectious particles are produced in L cells, a kinetics assay was conducted (Fig 11). As expected, no increase in virus titre was observed in wells inoculated with PV-3, a virus unable to infect L cells. By contrast, the virus titre increased after 2 and 5 days of incubation in wells infected by EV-D111, thus demonstrating the completion of the EV-D111 cycle in L cells in spite of the absence of any visible cytopathic effects.

Finally, we also evaluated the ability of EV-D111 to infect non-mammalian cells by inoculating C6/36, a cell line from *Aedes albopictus* that is used to cultivate arboviruses. No EV-D111-induced cytopathic effect was observed on this cell line (Fig 8) and no replication was detected through molecular screening (Fig 10).

## Discussion

Enteroviruses D are still enigmatic by many aspects. For decades, only two EV-Ds (EV-D68 and EV-D70) were known, which together shared very few phenotypic and epidemiologic features. They were only linked by their close genetic relationships in the capsid-encoding region, which led to their inclusion within the same species, then called *Human enterovirus D*. All three additional EV-D types discovered since the 2000s (EV-D94, EV-D111 and EV-D120) were found or detected in stool samples, which suggest their enteric tropism. This is supported by the fact that EV-D111 (in this study) and EV-D94 (in previous studies [7, 33]) were found resistant to acid treatment. Since EV-D70s are also resistant to acid pHs and are phylogenetically closely related to EV-D111 and EV-D120 in the capsid, it is probable that EV-D70 strains responsible for acute haemorrhagic conjunctivitis in humans derive from an ancestor that had an enteric tropism. As other known EV-Ds, EV-D111s do not have the additional short uORF recently described for some EV-As, Bs and Cs. The product of this uORF was shown not to be crucial for the replication of E-7 but improves the growth of this virus in gut epithelial cells [28]. The fact that the very beginning of the uORF is conserved among EV-Ds could indicate that a common ancestor of the current EV-Ds had a functional uORF that was subsequently lost; alternately, the conservation of this short stretch of nt could only be due to structural constraints that exist to properly fold this part of the genome, which is involved in genome translation [34].

Our phylogenetic analyses in the non-capsid genomic regions suggest that EV-D94 and EV-D111 evolve through intertypic recombination, a common feature of EV of species A, B and C [35–38]. This observation suggests that strains of the two types circulate in overlapping geographic areas and are able to co-infect cells. We did not observe any evidence of genetic exchanges between these two types in the one hand and EV-D68 in the other hand, despite the huge number of EV-D68 sequences that have been obtained during the EV-D68-associated outbreaks in the 2010s. Yet, the ability of recombination to give rise to functional chimeric EV-D68/EV-D94 genomes was previously demonstrated [33]. Our observation could be biased by the low number of EV-D94 and EV-D111 strains or by the fact that most EV-D68 sequences were obtained from strains caught in continents other than Africa. Nonetheless, genetic exchanges between EV-D68 and other known EV-Ds are probably impeded by their distinct tissue tropisms.

In this work, we did not address the receptor usage of EV-D111. The fact that an anti-CD155 antibody does not protect L20B cells against EV-D111 infection could indicate that this virus does not use CD155; however, it is not possible to rule out the fact that this antibody interacts with CD155 without interfering with the binding of EV-D111 particles on CD155. Nonetheless, in a previous study [7], a polyclonal rabbit serum against CD155 also failed to protect L20B cells against EV-D94. The same study has also suggested that EV-D94 does not use neither CD55 (also known as Decay-Accelerating Factor), a receptor used by the other known members of the species EV-D (EV-D68 [17] and EV-D70 [39, 40]), nor other receptors used by EVs belonging to other species, such as ICAM-1 [41], the vitronectin receptor [42], the Human Coxsackievirus-Adenovirus Receptor (HCAR) [43] and the *α2β1* integrin [44]. Therefore, to date, the receptors used by EV-D94 and EV-D111 remain unknown.

The identification of EV-D111 both in human samples and in one sample from a wild chimpanzee raises questions about the natural history of this virus. Our phylogenetic analysis does not support the existence of two separate lineages of EV-D111, one circulating among NHP populations and one among humans, and suggests a recent zoonotic transmission of this virus. Similar observations were made for other EV types: others and we previously reported that EV types EV-A76, EV-A89, EV-A119 and EV-B107 contain both human-derived strains and strains from samples of wild NHPs living in remote areas of the rainforest in Central Africa [11, 45]. A recent review suggested a transfer of EV-D111 from humans to the infected chimpanzee [46]. We cannot rule out this direction of transmission (directly or indirectly, through water for instance) but we believe that the alternate hypothesis, i.e the anthropozoonotic transfer from animals to humans, is, at least, as credible.

First, the hypothesis of the animal origin of EV-D111 is consistent with the fact that another EV-D, EV-D120, was also detected in wild NHP samples from Central Africa and has never been detected in human samples [8]. It is also consistent with the proposed zoonotic origin of EV-D70, which seems to have emerged among humans in Africa in the 1960s [47, 48]. The recent emergence of EV-D70 in humans is supported by two main arguments: i- previous seroepidemiologic studies have detected EV-D70-neutralizing antibodies in sera from cattle, sheep, swine, dogs and chicken collected in Ghana, Senegal and Japan, of which some had been collected prior to the emergence of acute haemorrhagic conjunctivitis outbreaks in humans [49]; ii- according to phylogenetic studies, all known EV-D70 strains seem to derive from a recent common ancestor [50].

Second, as EV-D94 and EV-D111 induce strong cytopathic effects in L20B cells, frequent human infections by one of these two types would have led to numerous false-positive detections of poliovirus by members of the Global Polio Laboratory Network that use the WHO cell culture-based algorithm, which mainly relies on the L20B cell line to detect polioviruses. Our two laboratories in Central African Republic and in Cameroon are members of the Global Polio Laboratory Network and we have never faced a high rate of detection of non-polio EVs in L20B cells in spite of the annual screening of thousands of stool and environmental samples in the framework of poliovirus surveillance. This suggests that human infections by these two viruses are not common in Central Africa; in the mean time, EV-D111 was detected in a sample from a panel of 54 NHP stools [9] and EV-D120 was detected in 5 samples out of 139 NHP stools [8]. In other words, EV-Ds are rarely found in human stools, even in Central Africa, whereas EV-D111 and EV-D120 were repeatedly found in very small panels of stool samples of wild NHPs.

Furthermore, none of the most common EV types circulating among humans in Central Africa (CV-A13, EV-C99, CV-A20, CV-A24, CV-Bs or echoviruses of diverse types [11, 14, 51]) was detected in the panels of NHP stools in which EV-D111 and EV-D120 were found. Yet, NHPs are susceptible to many types of human EVs, as observed in several occasions [45, 46, 52–55]. In this context, it is difficult to understand why the human EVs transmitted to wild NHPs living in remote areas of the African rainforests with minimal human contacts would precisely belong to two types that have almost never (EV-D111) or never (EV-D120) been found in human samples.

Last, although EV-D68 was first identified in California, Africa seems to be the source of EV-Ds: EV-D70 emerged in Africa and EV-D94, EV-D111 and EV-D120 have never been isolated outside of Africa. EV-D94, EV-D111 and EV-D120 are seemingly geographically restricted to African habitats that host NHPs and they were discovered during the last decades. These observations are consistent with the hypothesis that these three types circulate in wild reservoirs leaving in Central Africa (presumably NHPs) and that EV-D94 and EV-D111 have emerged from NHPs recently.

The hypothesis that humans are not the main hosts of most EV-Ds could explain why only five types have been identified within the species EV-D, despite the fact that the isolation of members of this species in cell cultures does not seem to be challenging. Indeed, like EV-D111 in study, EV-D70 and EV-D94 previously demonstrated their ability to infect cells of different mammalian species [32, 56, 57], including the L20B cells that are widely used for poliovirus surveillance. Some coxsackieviruses A of species EV-A induce cytopathic effects on L20B cells but require a relatively long incubation period to reach the complete destruction of the cells [58]. By contrast, EV-D111 (in this study) and EV-D94 (previously [7]) were found to rapidly induce the total destruction of L20B monolayers. Thus, EV-Ds could be responsible for false-positive detection of polioviruses in cell cultures, particularly EV-D94 and EV-D111, which circulate in Nigeria and neighbouring countries: Nigeria is now one of the last countries where wild poliovirus still circulates [59] and intense poliovirus surveillance is currently conducted in this geographic area within the framework of the Global Polio Eradication Initiative.

Finally, we believe that important pieces of the puzzle are still missing to understand the natural history and the epidemiology of EV-Ds. For instance, two seroprevalence studies previously demonstrated the presence of anti-EV-D94 neutralizing antibodies in more than half of human serum samples collected in Finland, Scotland, Cameroon, Zimbabwe and South Africa [7, 60]. This result suggests an active circulation of EV-D94 in some European and African countries among humans but it is difficult to understand how a virus that produces obvious cytopathic effects on many cell lines and circulates (at least sporadically) both in sub-Saharan Africa and in Europe could be consistently missed by all the enterovirus surveillance laboratories worldwide and particularly by those of the Global Polio Laboratory Network that routinely use L20B cells to detect polioviruses; furthermore, recent environmental studies of EV circulation based on viral isolation or in-depth sequencing approaches did not show any evidence of EV-D94 in Europe [61–63]. New studies focusing on EV-Ds in humans and in herd or wild animals, in Central Africa and elsewhere, have to be conducted to get new insights on the circulation of EV-Ds, on their natural hosts and their genetic and phenotypic diversity. To facilitate such studies, one EV-D111 strain characterized in this work has been made available for the scientific community via the European Virus Archive (https://www.european-virus-archive.com), a non-profit organization [12].

## Supporting information

S1 Table Composition of culture media used for cell growth. (PDF)

S2 Table Predicted cleavage sites of the polyprotein of different EV-D111 strains. The residues that are not conserved among all known EV-D111 sequences are highlighted in red.
(PDF)

S1 Fig Phylogenetic tree based on the short VP1 region where the sequences of Nigeria-2017 and KK2640 overlap each other. (PDF)

S2 Fig Immunofluorescence assay with anti-CD155 antibody D171. As expected, fluorescence was observed on L20B and RD monolayers but not on L cells: L20B and RD cells express CD155 while L cells do not.
(PDF)
