# Supplementary material for: Genetic and phenotypic characterization of recently discovered enterovirus D type 111
Source: PLoS Negl Trop Dis. 2019 Oct 17;13(10):e0007797. doi: 10.1371/journal.pntd.0007797 (PMC6818792; doi:10.1371/journal.pntd.0007797)
Supplement: S1 Table — (PDF) [file pntd.0007797.s001.pdf]

Cell line	Component (Final concentration)	Provider	Reference
RD	Minimum Essential Medium Eagle<br>HEPES (10 mM)<br>Foetal Bovine Serum (5%)<br>Glutamine (2 mM)	Sigma<br>Gibco<br>Gibco<br>Gibco	M5650<br>15630-056<br>10270-098<br>25030-081
L20B	Minimum Essential Medium Eagle<br>HEPES (10 mM)<br>Foetal Bovine Serum (10%)<br>Glutamine (2 mM)	Sigma<br>Gibco<br>Gibco<br>Gibco	M5650<br>15630-056<br>10270-098<br>25030-081
HEp-2c	Minimum Essential Medium Eagle<br>Foetal Bovine Serum (10%)<br>Glutamine (2 mM)	Sigma<br>Gibco<br>Gibco	M5650<br>10270-098<br>25030-081
LLC-MK2	Medium 199<br>Heat Inactivated Horse Serum (1%)<br>Glutamine (2 mM)	Gibco<br>Gibco<br>Gibco	31150-022<br>26050-070<br>25030-081
MA104	Minimum Essential Medium Eagle<br>Foetal Bovine Serum (10%)<br>Glutamine (2 mM)<br>Non-Essential Amino Acids Solution (1X)	Sigma<br>Gibco<br>Gibco<br>Gibco	M5650<br>10270-098<br>25030-081<br>11140-035
L	DMEM High Glucose<br>Foetal Bovine Serum (10%)<br>Glutamine (2 mM)	Gibco<br>Gibco<br>Gibco	11965-092<br>26050-070<br>25030-081
D171 hybridoma	RPMI 1640<br>Heat Inactivated Foetal Bovine Serum (10%)<br>Glutamine (2 mM)	Lonza<br>Gibco<br>Gibco	BE12-167F<br>10500-056<br>25030-081
C6/36	Leibovitz's L-15 Medium<br>Foetal Bovine Serum (10%)<br>Non-Essential Amino Acids Solution (1X)	Gibco<br>Gibco<br>Gibco	11415-049<br>26050-070<br>11140-035
