# Supplementary material for: Genetic and phenotypic characterization of recently discovered enterovirus D type 111
Source: PLoS Negl Trop Dis. 2019 Oct 17;13(10):e0007797. doi: 10.1371/journal.pntd.0007797 (PMC6818792; doi:10.1371/journal.pntd.0007797)
Supplement: S2 Table — The residues that are not conserved among all known EV-D111 sequences are highlighted in red. (PDF) [file pntd.0007797.s002.pdf]

	NGB-06-107	TOK-230	BAN-06-150	OUP-05-059	Nigeria-2017
**VP4/VP2**	APILK/SPSAE	APVLK/SPSAE	APVLK/SPSAE	APVLK/SPSAE	not sequenced
**VP2/VP3**	NAITQ/GVPTY	NAITQ/GVPTY	NAITQ/GVPTY	NAITQ/GVPTY	not sequenced
**VP3/VP1**	PDIGQ/SKILP	PDIGQ/SKILP	PDIGQ/SKILP	PDIGQ/SKILP	not sequenced
**VP1/2A**	SLTTR/GPGFG	SLTTR/GPGYG	SLTTR/GPGFG	SLTTR/GPGFG	SLTSK/GPGFG
**2A/2B**	EAMEQ/GITDY	DAMEQ/GITDY	DAMEQ/GITDY	DAMEQ/GITDY	EAMEQ/GITDY
**2B/2C**	YVIKQ/GDSWL	YVIKQ/GDSWL	YVIRQ/GDSWL	YVIRQ/GDSWL	YVIKQ/GDSWL
**2C/3A**	EALFQ/GPPQF	EALFQ/GPPQF	EALFQ/GPPQF	EALFQ/GPPQF	EALFQ/GPPQF
**3A/3B**	FAGMQ/GPYTG	FAGMQ/GPYTG	FAGIQ/GPYTG	FAGIQ/GPYTG	not sequenced
**3B/3C**	TAKVQ/GPGFD	VAKVQ/GPGFD	TAKVQ/GPGFD	TAKVQ/GPGFD	not sequenced
**3C/3D**	FTGKQ/GEIVS	FTDKQ/GEIVS	FTDKQ/GEIVS	FTNKQ/GEIVS	not sequenced
