# Supplementary figures and images for: Genetic and phenotypic characterization of recently discovered enterovirus D type 111
Source: PLoS Negl Trop Dis. 2019 Oct 17;13(10):e0007797. doi: 10.1371/journal.pntd.0007797 (PMC6818792; doi:10.1371/journal.pntd.0007797)

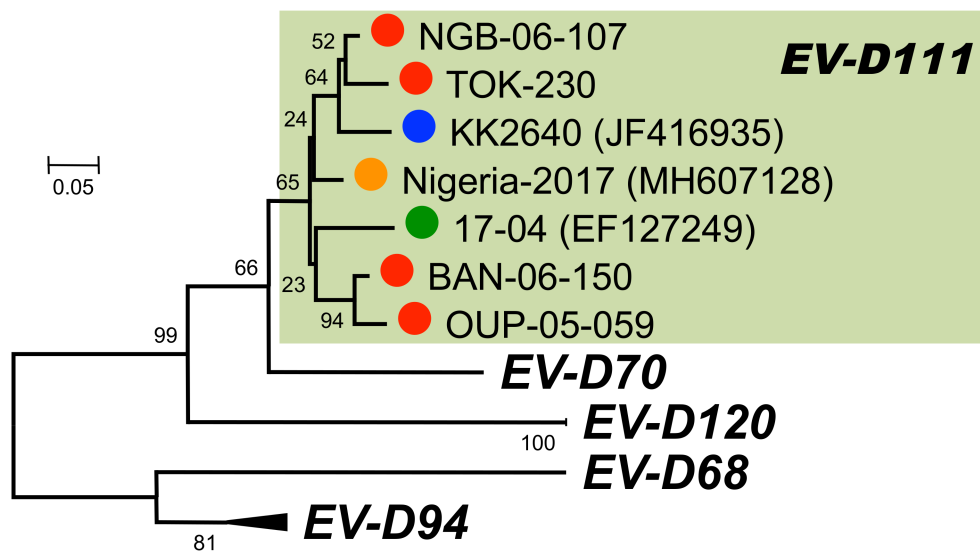

Supplement: S1 Fig — (PDF) [file pntd.0007797.s003.pdf]

**Visible**

**Fluorescence**

**L20B**

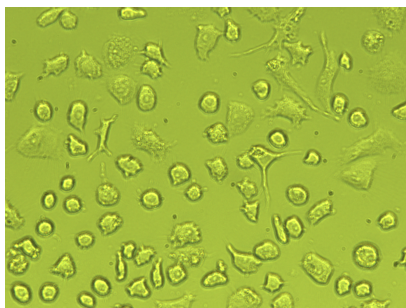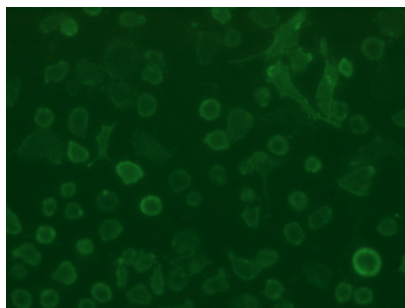

**RD**

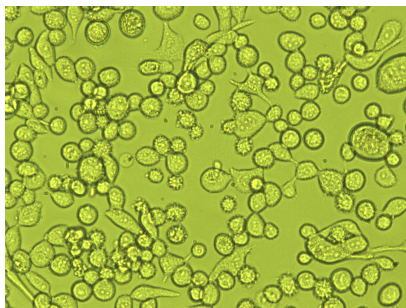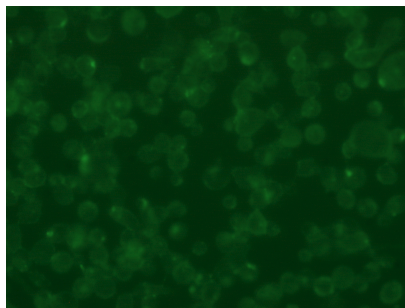

**L**

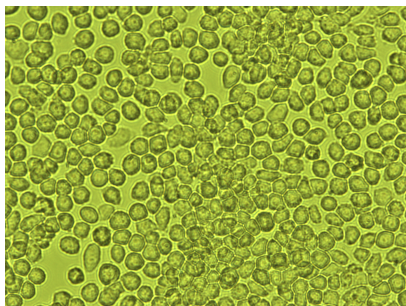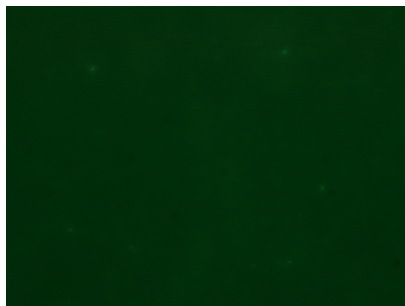

Supplement: S2 Fig — As expected, fluorescence was observed on L20B and RD monolayers but not on L cells: L20B and RD cells express CD155 while L cells do not. (PDF) [file pntd.0007797.s004.pdf]
